# Occurrence of abscesses during treatment with pazopanib in metastatic renal cancer: a case report

**DOI:** 10.1186/s13256-019-2318-y

**Published:** 2020-01-11

**Authors:** Ivana Puliafito, Alessio Russo, Dorotea Sciacca, Caterina Puglisi, Dario Giuffrida

**Affiliations:** 1 Department of Experimental Oncology, Mediterranean Institute of Oncology, via Penninazzo 7, Viagrande, I-95029 Catania, Italy; 2 IOM Ricerca srl, via Penninazzo 11, Viagrande, I-95029 Catania, Italy

**Keywords:** Pazopanib, Abscesses of lung metastases, Drug-related adverse effects, Metastatic renal cancer

## Abstract

**Background:**

Pazopanib is a multitarget tyrosine kinase inhibitor used in the treatment of renal cancer and soft tissue sarcoma. Its use is commonly associated with a number of side effects, such as hemorrhagic diathesis, neutropenia, leukopenia, thrombocytopenia, nausea, vomiting, abdominal pain, increased serum aspartate aminotransferase, increased serum alanine aminotransferase, decreased serum glucose, increased serum bilirubin, decreased serum phosphate and magnesium, fatigue, hypertension, diarrhea, anorexia, proteinuria, and hypothyroidism. Abscesses of metastases caused by pazopanib administration are rarely reported in the literature.

**Case presentation:**

We report a case of abscesses of lung metastases related to pazopanib in a patient with metastatic renal cancer. The patient was a 53-year-old Caucasian man who developed abscesses of lung metastases during the first 3 months of treatment with pazopanib. The abscesses resolved after 1 month by stopping pazopanib and administering adequate antibiotic therapy.

**Conclusions:**

We conclude that abscesses of metastases could be a rare side effect occurring during treatment with pazopanib in patients with renal cancer.

## Introduction

Pazopanib (Votrient; Novartis, East Hanover, NJ, USA) is a tyrosine kinase inhibitor used to treat kidney cancer and soft tissue sarcoma (STS). This drug is a multikinase inhibitor that has been demonstrated to inhibit vascular endothelial growth factor receptors (VEGFR-1, -2, and -3), platelet-derived growth factor receptor-α and -β, fibroblast growth factor receptor-1 and -3, and c-KIT. This inhibition affects tumor growth and inhibits angiogenesis, thereby slowing or stopping cancer cell proliferation and spread of malignancies. Furthermore, pazopanib binds to various physiological receptors and ion channels, such as histamine, opioid, serotonergic, dopaminergic, cholinergic, glutamate, adenosine, and adrenergic receptors and calcium, potassium, and sodium ion channels. Common adverse effects of pazopanib include headache, loss of appetite, weight loss, nausea, vomiting, diarrhea, erythrodysesthesia, changes in hair or skin color, and joint or muscle pain.

The efficacy and safety of pazopanib were evaluated in a phase III randomized, double-blind, placebo-controlled trial [1] that enrolled 435 patients with locally advanced or metastatic renal cell carcinoma. The study results indicated that Votrient significantly prolonged progression-free survival in comparison with placebo, both in the overall study population and in the cytokine-pretreated patients. The median progression-free survival was 9.2 months for patients who received Votrient and 4.2 months for patients in the placebo arm. Regarding safety, the study highlighted that most of the treatment-emergent adverse events were grade 1 or 2, even though grade 3/4 hypertension and diarrhea were observed with appreciable incidence and four pazopanib-treated patients had fatal adverse effects.

The efficacy and safety of pazopanib were evaluated also in patients with metastatic soft tissue sarcoma (STS) that achieved progression despite previous standard chemotherapy [1, 2].

In this report, we describe a case of abscesses of lung metastases related to pazopanib in a patient with metastatic renal cancer.

## Case presentation

A 53-year-old Caucasian man was admitted to our hospital with back pain in March 2016. He was a heavy smoker with a 30-pack-year smoking history. He had had hypertension for 2 years. Regarding his medical history, there was nothing in particular to note apart from metastatic renal cancer and its complications. Indeed, on physical examination, the patient appeared well.

The patient underwent computed tomography (CT), which showed a mass in the left kidney (75 × 53 × 105 mm), as well as lung and liver metastases, confirmed by magnetic resonance imaging examination.

He underwent hepatic biopsy with a negative result because of exiguity of the sample. The patient received left radical nephrectomy and splenectomy in April 2016, and postoperative histopathology revealed clear cell carcinoma of the kidney. In May 2016, the patient was admitted to our hospital again because of kidney failure and electrolyte alterations.

**Fig. 1:**
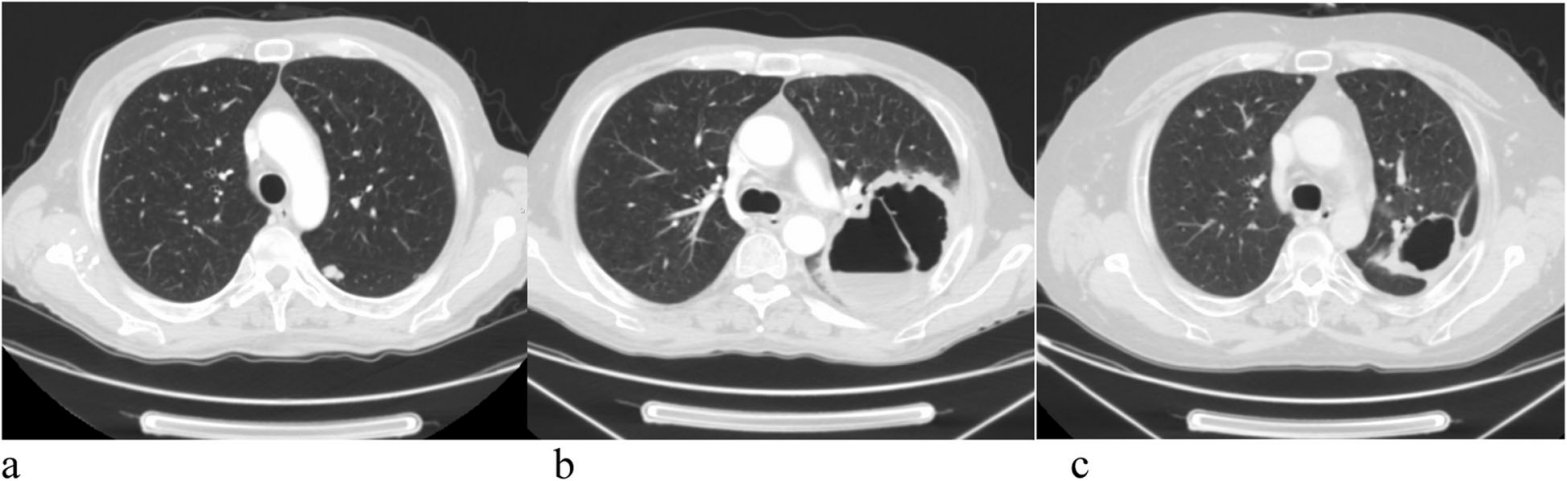
**a** Basal computed tomography. **b** Computed tomography slice showing necrotizing metastases with cavitation on lung similar to abscesses after 3 months of treatment. **c** Computed tomography slice showing resolution of abscesses 1 month after stopping treatment with pazopanib

A CT scan at that time confirmed lung and hepatic metastases, and the patient received supportive care with resolution of metabolic alterations (Fig. 1a).

In June 2016, he started treatment with pazopanib 800 mg daily for metastatic renal cancer.

After 3 months, he had a repeat CT scan (Fig. 1b), which revealed evidence of necrotizing metastases with cavitation on the lung similar to abscesses in the absence of fever. He stopped pazopanib and started therapy with antibiotics as suggested by a pneumologist.

One month after starting antibiotics, a CT scan showed resolution of the patient’s abscesses but progressive disease (Fig. 1c).

## Discussion

Pazopanib is a multitargeted tyrosine kinase inhibitor that principally inhibits VEGFR, producing block of tumor angiogenesis and growth. Pazopanib is currently approved for the treatment of renal cell carcinoma and advanced STS [3].

Our patient had a rare case of necrotizing lung metastases with cavitation induced by pazopanib.

Cases of abscesses have been reported in patients with STS treated with pazopanib [4].

Pulmonary abscess is a cavitation containing pus and products of necrosis with perilesional inflammatory area of variable dimensions. Common bacteria detectable in pus include *Streptococcus* spp.

In our patient, a possible mechanism of abscess origin could be necrosis of metastases because pazopanib works by decreasing the blood supply to the cancer. Suppuration of lung metastases could be secondary to bronchial obstruction or to superinfection of the central area of metastases not receiving blood. This process causes necrosis.

Another possibility is immunodepression in patients with malignancy.

The adverse event in our patient required interruption of pazopanib because consequences could be severe, including creation of communicating cavitations, pleural empyema, hemoptysis, diffusion of infection with migration of septic embolus, and chronicity of abscess.

In 2014, a single-center case series by Verschoor and Gelderblom was published [5]. They reported 6 cases of pneumothorax among over 43 patients with STS treated with pazopanib in their center. Pneumothorax was reported as an adverse event with a percentage of 3.3% in the phase III registered trial [2] supporting use of pazopanib for locally advanced or metastatic nonliposarcoma STS after prior treatment with doxorubicin and/or ifosfamide. In that study, the percentage of pneumothorax was 14%, superior to that reported in a registered trial. The six patients who developed pneumothorax during or after pazopanib therapy had subpleural lung metastases or pleural metastases. After the start of pazopanib treatment, patients had necrotizing lung metastases with cavitation or malignant pleural effusion [5]. No case of pneumothorax was reported in patients with renal cell carcinoma receiving pazopanib.

Pazopanib was tolerated in most clinical studies. The most commonly reported adverse events in patients with renal cancer treated with pazopanib were fatigue, diarrhea, and hypertension [6–10].

The relationship between pazopanib treatment and pulmonary abscesses can be supported by regression of the abscesses after interruption of treatment and antibiotic therapy.

## Conclusions

We suggest that abscesses that develop after pazopanib administration should be considered a rare toxicity after excluding other causes. Pazopanib should be stopped according to the severity of the clinical situation.

## Data Availability

The datasets created during and/or analyzed during this case are available from the corresponding author on reasonable request.

## Abbreviations

CT: Computed tomography
STS: Soft tissue sarcoma
VEGFR: Vascular endothelial growth factor receptor
